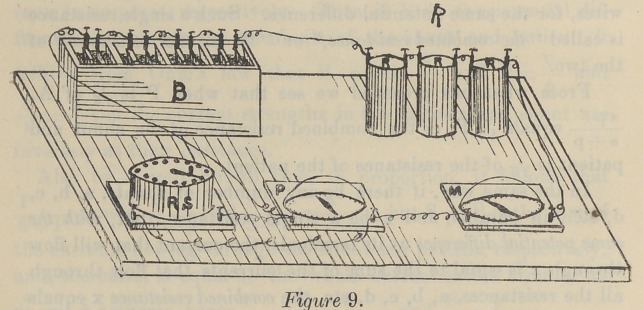# Discussion on Cataphoresis

**Published:** 1897-03

**Authors:** 


### PROCEEDINGS.

### Discussion on Cataphoresis.

Discussion of papers by Drs. Price, Barber, Hersh and Husted, Ohio State Dental Society, December, 1896.*

DR. L. E. CUSTER, Dayton: We have, in these four papers, had more good matter on cataphoresis presented than I have heard in any Society either East or West.

Dr. Husted has presented a paper somewhat elementary in character, but nevertheless what is needed for us as busy dentists. The applications of electricity in dentistry are becoming so numerous that the time is at hand when we must give a little attention to electricity.

The illustration of the meaning of the volt and the ampere is good. In the arrangement for quantity of current in amperes we may consider the vessel as lying upon its side. The pressure of the water is not very much, but a large quantity of water would flow out if a side were removed. If the vessel be placed on end, and the water be allowed to escape at the lower end, then there is not so much water escaping, but the pressure is increased. This would represent the volt in electricity. The *ampere* is the current and the *volt* is the pressure.

*These papers appear in February No. of DENTAL REGISTER.

Now, in this illustration, about the same amount of work will be done by this water in escaping, whether the vessel is laid on its side or stands on end. This is represented in electricity by the term *watt*. The product of the volts multiplied by the amperes and 746 watts make one-horse power.

In referring to the peculiar current, once obtained by the author of the paper for sensitive dentine, it seems strange that it can not be reproduced, and it seems to me I would not stop until it was, because that is the ideal we have long sought. The method of a gentleman from the northern part of our State savors somewhat of quackery. It has been ascertained that the method of obtunding dentine, if it may be called such, with the galvanic current alone, is not a success.

Dr. Hersh is correct when he states that dentists are not electricians. This process of cataphoresis requires more breadth of knowledge than any process in dentistry. It requires not only a knowledge of the ordinary operations of electricity, but a careful study of the principle under which it operates, and the conditions necessary for a successful application. We must know something of electrolysis and resistance, and the control of our current. I have favored the use of the 110-volt Edison current, because in my own city it is quite satisfactory. I get my current direct from the main line and am not annoyed by any great fluctuation in voltage, which it has been my experience to meet with while operating in other cities. It has some dangers and faults, but gradually we are finding them out. Only a short time ago I could not account for a sudden shock to a patient, but upon examination I found that she had touched the iron-work of the chair, which made a " ground " through the water flowing in the rubber tube of the fountain cuspidor.

In operating with the Edison 110-volt current, in various portions of the country, I have noticed that there was a great variation in the currents in different places. In some there was so much pain in the application of the current that the operation could not be called a success as far as being painless was concerned. When the appliances were perfectly quiet the patient would, from time to time, experience sudden and severe shocks of pain, much as if the current had been broken. I feel safe in saying that in these instances, where there was intermittent pain to the patient, and the electrode was being held quiet, it was due to the fluctuation in the main current. The fluctuation is usually a lowering of the voltage, and so quickly done that it is not noticeable on the ordinary volt-meters in use. It is not a drop of a few volts only, but quite a number; at times, perhaps, twenty or thirty.

Dr. Molyneaux cited a case where he thought there was a short circuit produced by two outer wires coming in contact. If this really occurred, it would raise the voltage only three or four volts, yet that would be enough to give the patient a shock. When one is using a shunt, however, there is not the danger there is from using a series current, even if it is the Edison. The current obtained directly from the wire passing the building is the most constant and satisfactory. A cataphoric appliance, operated from the same wires that supply elevators or machinery, or where they supply many lights, is not very satisfactory, because, when any one of these is cut in or out, it varies the voltage used for cataphoresis.

There is one thing in favor of storage-batteries, and that is that they are active. The elements are always ready for an opportunity to combine, and you are sure of a constant current. The dry-cell battery seems to warm up for a time and then lets down.

In regard to electrodes, the negative should be of sufficient sise not to cause a blister under it. If it be large there will be less reddening of the tissues. One of the principal points of success in cataphoresis is that the positive electrodes must be held quiet.

In the treatment of proximal cavities, I make a sort of hook of platinum wire and hang it between the cavities to be operated upon, and pack the saturated cotton about it.

The poles should be placed as near together as possible, for by so doing you get less pain when applying the current, because less voltage is required.

I would advise all dentists who are using cataphoresis for the first time, to take some simple cavity where all walls are visible, and in some location easy of access.

Dr. Barber began his paper by stating that there was a difference in the susceptibility of patients to the electric current. This, I believe is, in a measure, correct; at least, I find that, under similar conditions, some patients will feel the current more than others. But in cataphoresis we use so little current that I am inclined to the belief that it is not all due to the peculiar electric pain. It is probably the same thing that we observe every day; some patients seem indifferent to the cutting of the same kind of dentine that may be excruciating to others.

In regard to the percentage of solution, I have always held that it is cocain that we are trying to project into the tooth, and if our solution is all water, that is what we will have, and nothing else; but if it is cocain and a saturated solution, we will then drive cocaine into the dentine. There have been so many solutions of various percentages presented that you will probably comply with somebody's formulæ, whatever percent you use. The cavity should be kept well flooded for two reasons—one, that there will not be a waste of energy, and the other that there will not be a shock by the variation of resistance to the half-moistened cotton.

I have found no virtue in guaia-cocain over pure cocain, and the odor of the former is a decided objection. It is also a poor conductor as compared with pure cocain.

The essayist was correct in stating that the current flows through the tubuli of the dentine. You will get anæsthesia only where the mouths of the tubuli are exposed in the cavity, unless the process is carried far enough to penetrate the pulp, and anæsthetize that and the dentine whose tubuli are covered with enamel will be anæsthetized reflexly. In the case of the molar, the dentine of the buccal cavity was anæsthetized after the pulp had been affected through the exposed dentine in the crown.

The paper of Dr. Price is one of the best yet presented on cataphoresis, and although it is scientific and somewhat technical, it is presented in such a way that it is within the understanding of all. When it appears in print every one should study it carefully and use it for future reference. The objection to the water and all forms of series rheostats is well taken, for there are serious objections to their use in cataphoresis. We have here had a clear illustration of the direct and shunt current, and also an explanation of the deceptive term " volt-selecter." A volt-selecter is simply an instrument which gives a shunt current for cataphoric and other purposes. It is convenient because it can start with zero and increase in voltage at will, by simply shunting or switching a little of the main current on a side track. In England the railroad switch is called a shunt. It is safer, because the patient is not in the main current. I have had something to do with the making of cataphoric appliances, and in making one for the 110-volt current I put in a third shunt-coil simply as a matter of safety, and in so doing I noticed there was less shock to the patient than where this coil was left out, and so have continued to use it. Dr. Price has told you why this is so.

I stated, before the Mississippi Valley Society, that the best position for the negative electrode was on the cheek or as near the positive pole as was practical. I had no especial a reason for that, except that it required a higher voltage to do the same work when the cathode was held in the hand than when held on the cheek, and the lower the voltage the less would be the pain. Dr. Price has illustrated that very nicely.

When we make measurements of voltage and amperage in dentine cataphoresis, we must bear in mind that the resistance of the dentine is the greatest in the course of the current from the positive to the negative (of course, enamel is not considered, because it is a non-conductor). For this reason the area of exposed dentine and the distance to the pulp are the principal factors to be considered in the measurement of current. Abraded dentine will probably offer very high resistance as compared with freshly-exposed dentine.

Some of the appliances for use with battery are series instruments, and in order to increase the voltage it is often necessary to go back and throw in additional cells. This is a loss of time as well as a more painful way. It requires about one-eighth more cells to give a shunt current instead of a series current, ordinarily. The cost of this extra amount fully repays, because it saves time; it enables the current to increase from zero, and being a shunt the current is less painful.

Dr. J. R. Bell, Cleveland: In my practice I have found it necessary to make a few preparatory steps before using cataphoresis on my patients. The first is to give the patient information regarding the cataphoric appliance and the effects of the current, and in this way not only educate the patient, but establish a confidence that is very essential for the best success in any operation. The next thing is to ascertain the condition of the teeth to be operated upon. There are two or three conditions where we will not get satisfactory results unless they are modified. One is the presence of inflammation in the dentine. Where this condition exists, I have found it advisable to first reduce the inflammation in order to get the satisfactory results desired. In the teeth of smokers we sometimes find a dense lining in the tooth cavity, which must be removed before we obtain a free flow of the electric current through the tubules of that tooth. I have had the best results from the use of a saturated aqueous solution of cocain, and in cases where, for instance, two molar teeth were decayed and cavities approximating, I have obtunded both, by filling in between with saturated cotton and applying the current to one of them.

Where teeth have been previously treated with creosote or carbolic acid, I have not been able to get as satisfactory results as where such agents have not been employed. Another thing: I do not pay any attention to the current passing through the clamp, for I first apply the rubber-dam, then adjust the clamp over this, and I know that it is then insulated. I have not yet in my practice found it necessary to insulate fillings already in the teeth.

J. R. Callahan, Cincinnati: It seems to be hard for many dentists to get electrical terms well fixed in mind. The best plan that I have found is to compare the electrical current with a dam and mill-race. The dam represents the resistance of the ohms; the water above the dam represents the pressure or the volt; the escape over the dam represents the current in operation or the ampere; and the mill-race represents the shunt.

There is a number of points in the papers that it is necessary to remember; one is the necessity of having an absolutely-constant current. Another is the resistance we have to contend with. Dr. Price in his paper gave the resistance of the body at from 20,000 to 70,000 ohms. This statement greatly impressed me, and also that of the difference in resistance in different parts of the same cavity, and in cavities kept moist and those that become dry. Tooth substance offers more resistance than any other tissue of the body. Taking into consideration, however, that the resistance is lessened the nearer we approach the pulp of the tooth, I generally excavate as much as possible before applying cataphoresis, and it is astonishing how many I finish entirely without finding it necessary to use the current at all.

You all realize the necessity of keeping the electrode in constant contact with the tooth-substance or moistened cotton in the cavity, for you get a shock at the breaking of the current. This is apt to occur where the electrode is held in the hand by the operator, and to overcome the objection I take a piece of gutta-percha, warm it, and place on the clamp and pass the platinum electrode through this. It holds the electrode in the cavity and you are sure of constant contact, and it also gives one the free use of his hands. Some, it seems, have taken offense at the remark Dr. Price made regarding there not being a perfect instrument yet on the market, but I think that is true. If I were going out with an instrument I would have a storage-battery, and a volt and ammeter-attachment, and a water or shunt resistance. No appliance is complete without a pole-changer in connection.

Dr. Henry Barnes, Cleveland: One writer said that he used a large negative electrode, instead of a small one. I have tried the large electrode, but now use one smaller than a silver dollar. I have had difficulty in getting constant current through by attaching the positive electrode to the clamp, and find that the result is more satisfactory when the electrode is held with a steady hand. I have used alcoholic solutions of cocain, but not in the manner suggested by Dr. Barber. Where alcoholic solutions are used, the anæsthesia is of less duration than when the solution is an aqueous one. Some months ago I made the statement that the oral fluids used in connection with the cocain solution would produce anæsthesia, even if the rubber-dam was not used. When using cataphoresis in that way, however, I do not use a high voltage for fear of injuring the tissues. Dr. Jack advises the citrate of cocain. I tried this preparation and got no results at first; but when I used it in connection with the oral fluids as a solvent, I obtained anæsthesia of the dentine in from five to eight minutes, where otherwise the time required was from ten to fifteen minutes. This short time, though, is not always the case. Where a cavity proximates a filling in another tooth, I cut a narrow strip of photographic film, smear one side of it with gutta-percha solution, run it through between the teeth, allowing the dry and smooth side to come next the cavity to be filled. The gutta-percha sticks the film to the other tooth and completely insulates the filling. With the current that I am using I have found it necessary to test for polarity before each operation, as the currents are at times reversed.

Dr. J. W. Clark, Louisville: One case that recently came under my observation was that of a pulp to be extracted. Another dentist had endeavored to kill the pulp with an application, but was not successful. I applied a 20-percent solution of cocain, cataphorically, and got immediate results, so that inside of eight minutes I was enabled to extract the entire pulp with a broach, and without pain to the patient. Another case was two proximal cavities in the central incisors, which were so sensitive that even the insertion of cotton caused pain. Applied cataphoresis ten minutes, when I was enabled to prepare both cavities as far as the retaining pits. A second application of four minutes enabled me to prepare the remaining portions without pain. I had the same results when applying to proximal cavities in bicuspids. I have not had good results from the application of cataphoresis to inflamed pulps.

Dr. A. L. DeVilbiss, Decatur, Ind.: I can cite a case where the pulp was inflamed from having been treated with arsenic, but the application of cataphoresis made it possible to extract it without pain. Another case that was a good test for cataphoresis was that of a lower molar, badly worn with mechanical abrasion; so sensitive that the patient could scarcely eat. There was a small cavity on the proximal side of the tooth. Made application of cataphoresis, and the results were excellent. After preparing, I touched the surface with chlorid of zinc, then filled, and the patient has experienced no further trouble.

Dr. O. N. Heise, Cincinnati: In regard to the treatment of inflamed pulps and their extraction from the pulp-canals, would say that I have done so successfully by means of the electric current. My method is to use, at first, a very mild current, say of two volts (so mild as not to cause any pain), with the cocain, seeing, however, that the electrode does not come in direct contact with the pulp, but have plenty of cotton intervening, using the positive pole, and, in some cases, have succeeded better by using both poles, and alternately changing the flow of the current by reversing the poles; let it act until you have relieved the hyperæmia of the pulp, then gradually increase your current until it has thoroughly anæsthetized the pulp, after which, by means of a fine broach in the handle of the electrode-holder, insert broach alongside of the pulp, having, however, reversed the current, using the negative instead of the positive pole; allow it to act for a few moments, until it has decomposed the end of the pulp, which action is shown by the appearance of a froth around the broach. Just how long to leave the negative pole in contact with the pulp is a matter of experience; do not leave it too long, as the negative pole is caustic in its action, and might bring about some pericementitis, although I have not observed it. When sufficiently decomposed, by a little manipulation the pulp can be extracted as a whole, without a drop of blood, leaving the canal in a clean, aseptic condition, ready to fill at once.

Dr. W. A. Price, Cleveland: I see Professor Neiswanger, of Chicago, is here. I am sure the Society will be pleased to hear from him on this subject.

Dr. C. S. Neiswanger, Chicago: Let me assure you that I fully appreciate the honor of being called upon to address this large assemblage of the representative dentists of Ohio, and as there are several important papers yet to be read, and the time is growing short, I will not detain you long.

That electricity is a therapeutic agent of undoubted value is a fact concurred in by every branch of the medical and dental professions, but in times past so little was understood as to its physical laws or therapeutic action that it seems almost impossible to elevate it from the domain of charlatanry to its proper place. For this very reason I was much pleased that a majority of the papers treating on the uses of electricity in dentistry were from a rudimentary-physical standpoint, which shows a start in the right direction, for, let me assure you, that it is only by a study and knowledge of its physical laws that we can obtain correct therapeutical methods. You can not obtain the summit in company with the mob of charlatans who have used electricity as a cloak of learning these many years, but you must do it alone. A great reformer does not carry the crowd with him, but leaves behind facts which induce them to follow.

In listening to these papers on cataphoresis and the discussions following them, I have been delighted to note the absence of either enthusiasm or pessimism. That is as it should be. The enthusiast hurts the remedy and injures himself; while the pessimist, with his narrow prejudices, builds around himself a wall which, in time, he will not be able to surmount.

I am not a dentist, and as "the shoemaker should not judge above the last," I will confine my remarks on cataphoresis to facts which my own experience has proven.

Because a constant current goes from positive to negative, it is generally supposed that all medicaments must be placed upon the positive pole, so as to be *forced*, as it were, into the tissues by the *direction* of the current alone. Such is not the case, however, for cataphoresis is an electrolytic process, and in every instance the medicament is broken up into its elements, some of them going toward the negative pole and some toward the positive.

In the nomenclature given us by Faraday, those ions or products of decomposition which appear at the anode or positive pole, he called "anions," and those which appear at the cathode or negative pole, "cations."

The anions are electro-negative, and are repelled by the negative pole because they are the same potential as that pole. The cations are electro-positive, and being repelled by the positive pole, whose potential is the same, they are drawn to the negative pole in accordance with the well-known law that "unlike poles attract each other."

Iodin, bromin, chlorin, oxygen, etc., are anions or electro-negative elements, and have a strong affinity for the positive pole; therefore, when treating, for instance, an enlarged gland with solution of potass. iodid, we must use the solution on the *negative* pole if we wish to utilize the resolvent effects of the iodin. All of the metals, so far as we know, are "cations," and appear at the negative pole; if, then, we put a solution of potass. iodid on the *positive* pole and complete the circuit through some conducting medium, the potassium hydrate, being a metal and a cation, will be transferred through the medium to the negative pole, while the iodin, being an anion, *will remain at the positive pole for which it has an affinity*, and we will merely have the same effect as from a local application of iodin. All the bases are electro-positive or cations, and if we were using a solution of morphia sulph. or cocain hydrochlorate, we would apply from the *positive* pole, when the base, which, in this instance, we wish to utilize, will be transferred through the tissues toward the negative pole, for which it has an affinity. When using a solution of potass. iodid it is not the purpose of the operation to convey the potassium or base through the underlying tissue, but the iodin.

In my first experiments with the so-called cocain cataphoresis for obtunding sensitive dentine, I used a 50-percent solution on the positive pole and had excellent results; then I used a solution of 30-percent, 20-percent, 10-percent, and when I finally discovered that I could produce just as complete anæsthesia with a 2-percent solution, I began to look around for some other cause for the anæsthesia than the cocain; and to-day I believe it is due more to the *polar action of the current* than to the medicament we use. For aconite on the positive pole of a constant current has the same local effect as cocain, and it is my belief, although I have not tried it, that *a piece of wet cotton* attached to the positive pole and applied to a sensitive cavity *will do the work*. This may seem strange, especially to those who have not given the subject special attention, and I will give you my reasons:

The most definite and well-proven points regarding the *polar action* of a continuous current is that the positive pole is acid, sedative, and hemostatic. The negative pole is alkaline, producing a hypersensitive condition and increasing bleeding. Each is diametrically opposed to the other. Dentists just beginning the use of electricity will do well to keep these points in view, as success is largely dependent upon which is used for the active pole.

As far back as the year 1859, Funke discovered that a sound living nerve is neutral or feebly alkaline, but changed to acid on coagulation setting in, or on exhausting it by prolonged mechanical or electrical stimulation. The beginning of the death of the muscle is marked by a progressive acidity and subsequent coagulation of the muscular plasma. The same is true of nerve substance as well. Then, if it is true that the death of the muscle or nerve commences when an acid condition sets in, it is also true that an inflamed or over-active condition is due to alkalinity.

All inflammations are primarily local, due as stated above, to excessive alkalinity of the part, not because the system contains an excess of alkali, but that we have an unequal distribution of probable normal alkalinity. We almost fear to state how we believe this pathological condition is brought about, lest we be charged with being too ultra in our deductions; but a few years hence a person may not be considered a "crank" who advocates that it is due to a disturbance of the normal electrical currents traversing the body.

We are glad to quote, in this connection, from such eminent authority as Dr. J. Mount Bleyer, who says: "Yet all this points to the one conclusion and the one deduction, that animal electricity comes first; that it is the prime factor in all the processes of change, of chemical action, or otherwise, within the living body. That without its stimulus of polarization, no chemical action can be called into life, consequently none can go on, and tissue metamorphosis, which is life itself, must cease."

Why is it, then, when we place the positive pole over an inflamed and painful surface that the inflammation and pain subside? Oxygen is set free at the positive pole. Oxygen is an acidmaker, and the part in contact with this pole being changed to a condition of acidity, the tomporary death of the part has commenced, or is in a state of sedation, evinced by a circumscribed anæsthesia. But what has become of the alkalinity that existed previous to the application of the positive pole? It certainly has not been neutralized by the acidity of that pole, because that would necessitate an evolution of gas, which has not taken place.

Alkalies are electro-positive substances and have affinity for the negative pole. Consequently the excess of alkali at the point of inflammation is transferred to the neighborhood of the negative pole, which immediately assumes a hypersensitive condition, proving that excessive alkalinity causes inflammation, because the part was precisely normal before the application of the negative pole.

Because of the fact that alkalies are transferred to the region of the negative pole and produce a hypersensitive condition at that point is my reason for taking issue with one of the essayists who advocates placing the indifferent electrode on the cheek. The fact is, the larger the indifferent electrode (which in most dental operations is the negative terminal of the battery) the less irritation we have in the tissues underlying that electrode, because it is distributed over a large area, and as all the electricity that passes through the large indifferent electrode must also pass through the small active electrode within the cavity of the tooth, we can concentrate the *polar effect* at the positive terminal (in the tooth) and disseminate still more the irritating effect at the negative pole by *separating the electrodes as far as possible*, all of which teaches us two facts, viz.: (1). Not to use a small, indifferent electrode, and (2), not to place it upon the cheek, but upon the hand, or as far removed as possible from the active electrode.

In the excellent paper read by Dr. Price, he calls attention to the difference in resistance when the indifferent electrode is placed upon the cheek and when placed in the hand—the latter being much greater, but if the skin upon the hand were the same texture as that upon the cheek the difference in resistance would be inappreciable, because the resistance of the tissue underlying the skin is almost nil.

The so-called anæsthesia produced by a rapidly-alternating current is brought about in a different manner than with galvanism and is more the result of mechanical than chemical action.

The alternating current which comes into our houses for lighting purposes, has a pressure of 52 volts and alternates about 133 times per second, is a good sample of this manifestation of electricity; the rapid impulses given the muscle by this current brings on a tetanic spasm, which soon wears it out, causing it not only to lose its normal animal current, but to assume an acid condition, which, in this instance, is not due to polar action as with the positive of a galvanic or direct current, but by bringing on the temporary death of the part by fatigue from excessive and prolonged electrical stimulation.

I, therefore, believe it is possible to utilize this current for obtunding sensitive dentine, and taking into consideration that most of the smaller towns throughout the country are supplied with this character of current, it is worthy of your serious consideration and ought to be given a thorough test. The Jewell Graphite Rheostat, or in fact any good controller arranged on the shunt principle, will control this current as well as the 110-volt direct.

One word more and then I am through: Dr. Price has suggested to me that a milliampere meter, expressly for dental uses, would be an invaluable addition to secure definite results and reliable data in this class of work, and is of the opinion that the range of scale should be ten milliamperes subdivided into one-tenth milliampere divisions. It is, therefore, my intention to present the views of Dr. Price to Mr. E. W. Jewell, the inventor of the Jewell Standard Measnring Instruments, with the hope of being able, in a few weeks, to show you an instrument of this kind. (Since the above was put in type we understand that a milliampere meter of this kind has been constructed by Mr. Jewell, and is for sale by the McIntosh Battery and Optical Company, of Chicago.)

Dr. W. Buzzell, Port Clinton: I would like to ask the gentleman a question: Whether the accumulation of fat under the skin, where the negative electrode is placed, does not have its influence on the amount of resistance?

Dr. Neiswanger: Yes.

Dr. L. L. Barber, Toledo: How small an amount of current do you think will do the work of obtunding dentine?

Dr. Neiswanger: The milliameter should measure from three to five milliamperes, depending on the size of the cavity being treated—two or three milliamperes in a small cavity would do the same work as five milliamperes in a large cavity.

Dr. J. S. Cassidy, Covington, Ky.: I have been greatly pleased with the remarks of Dr. Neiswanger. It made me feel good when he said that cataphoresis is an electrolytic process. Perhaps his statement is a little dogmatic, but, yet, I have held that it is at least partially an electrolytic process. I was pleased to hear the doctor refer to the use of the negative pole in connection with certain medicaments, for that is just what I spoke about at the Missisippi Valley Dental Society last April, and inquired whether we should not use the negative pole instead of the positive. When we wanted to get the full effects of such medicaments as iodine, etc., but the quesion was not fully answered at that time. A long time ago I used to apply various medicaments electrically. With KI decomposed thus, there was no appreciable penetration of the freed iodine. This fact is not to be wondered at, when we understand that electrolytes are composed of electro-negative and electro-positive radicals, and that the laws of electrolysis must be obeyed. The negative radical iodine wants to stay with the positive pole, and, therefore, does not penetrate, but, if free iodine be used in *aqueous* solution, then penetration occurs. Why is that?

Dr. Neiswanger: After the iodine remains long enough to become the same potential it is repelled, and thus driven into the tissues. The more highly electro-positive the substances are, the deeper will they penetrate into the tissues. This process should be called cataphoresis and anaphoresis.

Dr. Cassidy: Yes; and might not the iodine be forced toward the cathode by reason of the more highly electro-negative oxygen set free simultaneously from the waters pre-empting the anode? Cocain hydrochlorid is a good conductor and decomposes into free HCl, and the alkaloid cocain; the alkaloid itself does not decompose, and, being electro-positive in its nature, tries to reach the cathode, or, in other words, to get as far away from the positive pole as possible.

Dr. W. A. Price, Cleveland: We dentists see things somewhat differently from the physicians. If cataphoresis is continued, through even a very thick layer of dentin, long enough we get a thorough anæsthesia of the pulp, which we could not do if the effect was produced solely by electrolysis. Suppose we wish only to anæsthetize the dentin, our pain-limit of the current which controls the amount is not decided by the sensation in the tissue we are anæsthetizing, but in another tissue in the path of the current, viz., the pulp. The physician does not have this condition, nor anything similar to it, and this entirely prevents comparison of methods. The idea of placing the negative pole at the greatest distance from the positive is all right if the action is only that of electrolysis, but if it is partly or largely osmosis that takes place, then it is different, and the negative pole should not be placed at such a distance. The more resistance you have to overcome the more pain there is produced with variations of current, so, if you want the least pain you must have the least resistance. To illustrate the above: If there are 60,000 ohms resistance in a tooth, and 10,000 in the patient elsewhere, then, the zone of neutrality of electric influence must be somewhere in the dentin of the tooth being operated upon, and the effect on all tissues beyond that, including the pulp, would be stimulating and not sedative, according to the theory of electrolysis only. If the end of the root is calcified, or something of that sort, it is a question whether we do not get dissipation of the current toward the gum and not through the end of the root.

Dr. J. F. Stephan, Cleveland: Dr. Barnes spoke of the use of citrate of cocain. I have used this preparation and have been well pleased with the results. I was surprised in one case where I used it in a cavity. There was a cavity approximating in the adjoining tooth and although I treated only one of the cavities, I found that the other had been desensitized also, so that I was enabled to excavate it clear to the grooving points. My method of applying cocain is a little different from that of others, so far as I know. I take the crystal or powdered cocain and apply it to the surface of the cavity, then place a piece of cotton moistened with water over the cocain in the cavity, then apply the electrode on the cotton. I have gotten satisfactory results in this way in less than five minutes. May it not be possible that the citrate of cocain is separated into its elements more readily than the hydrochlorate, and the apparent difference in time of its action be due to the difference in affinity of decomposition. For obtunding the pulp I think the citrate is superior to other preparations, and I like to use the crystals better than any aqueous solution.

Dr. H. F. Harvey, Cleveland: Where a cavity is partially opened and you apply cold water, it produces pain, so I have practiced warming the solution before placing it in the cavity, and find that it is an advantage. I warm the slab in water and then make up my solution on the warmed slab. By doing this you will save your patient considerable pain.

W. H. Hersh, Piqua: In regard to the fountain-spittoon accident that I cited in my paper, the water had been splashed over the cuspidor, so that when the patient accidentally touched the cuspidor with the back of her hand she got a "ground" I had previously warned her of the danger. For all I use the shunt current; patients are thus liable to get a shock, but it is nothing compared with what it might be if the 110-volt current were used direct. Taking these things into consideration I want to use a current that is reliable and yet not dangerous in case of such accidents, and, therefore, I do not use the 110-volt current. Dr. Gillett says he is not sure but that the battery is best. Storage-cells are best, perhaps, but it is so much trouble to keep them in order.

Some one spoke about a blister being produced where the negative electrode came in contact with the tissues. I have not used current enough to produce a blister, but have several times got a deep reddening of the parts.

Dr. J. Taft, Cincinnati: There are many things in regard to cataphoresis that should receive more consideration and study —as the varying susceptibility of patients to the electric current, decomposition of the medicament used. Is it always decomposed? The extent of penetration of the medicament; it is the presumption that it penetrates deeply, if so, how is it accomplished? Some say that the current carries the substances into the tissues, but this is thought not to be the case by some. There is not enough vascularity in the teeth to account for deep penetration, the tubules are already full of material, completely occupied, so it seems it would be with difficulty that deep penetration could be accomplished.

One speaker said that he first treated the dentin to reduce sensitiveness, then applies cataphoresis. This seems strange to me, to get a cure before applying that which you expect will cure. Does cataphoresis destroy the life of the dentin? There is an uncertainty about the efficiency of the process that should receive more attention. Many cases fail. Why is it, and where do these failures occur? Then, the conditions of the teeth to which the current is applied should be thoroughly studied.

Dr. H. G. Husted, Oberlin: I generally turn on a number of cells until the patient feels the increasing sensation, and then turn off one, which immediately relieves it. There is a great difference in patients. Some will stand twenty times the amount of current that others can.

Dr. L. L. Barber, Toledo: When I have cases where the teeth are excessively sensitive I place a temporary filling in the cavity, and when removed, what appears to be the normal dentin is found. Dr. Bell stated that where escharotics had been used we could not get the desired effect from cataphoresis. Teeth are bleached where arsenic has been used to destroy the pulps, and I have, after the application of arsenic to destroy a pulp and found it still sensitive, taken out that pulp without pain by means of cataphoresis and cocain.

Dr. Henry Barnes, Cleveland: I had a unique case some time ago. It was that of a cavity on the labial surface of a tooth where I had to hold up the rubber-dam with an instrument. Applied cocain cataphoretically until the cavity was completely desensitized, when the instrument I was holding slipped and the dam pulled away far enough to admit saliva, when sensitiveness immediately returned, so that when I touched the cavity with an instrument pain was felt by the patient. I thought that I had touched the gum instead of the dentin, but a second attempt was just the same, and I had to reapply the current before I could continue the work painlessly. What was the cause of this? Has any one else had a similar experience?

DR. HOUGHTON, Columbus: You were probably surcharged with electricity yourself, and when you used an instrnment on the tooth it caused a shock to the patient.

DR. W. A. PRICE, Cleveland: There is a condition involved in the problem of shunts, of which I had not time to speak, in my paper, and which is of considerable importance in the particular condition with which we have to deal. It is the relation of the total resistance of the circuit to the relation of the shunts themselves.

You remember we deducted as follows: Suppose a shunt around the patient. Let p represent the resistance of the patient, and a the resistance of the shunt, and S and P the currents in amperes, respectively. Then if V be the potential difference in volts at the terminals of the shunt and patient, it follows from Ohm's law that S equals $\frac{V}{S}$ and $P=\frac{V}{p}$ and $\frac{P}{S}=\frac{s}{p}$; or the current strengths in the patient and shunt are inversely as their resistance.

Also by a well known rule, in proportion, it follows that $\frac{P}{S+P}$ equals $\frac{s}{s+p}$ and $\frac{S}{S+P}=\frac{p}{s+p}$; but S + P is the sum of the currents flowing through the shunt and patient, respectively, and, therefore, is equal to the whole current in the circuit, let us say A amperes, hence, $\frac{P}{A}=\frac{S}{s+p}$ and $\frac{S}{A}=\frac{p}{s+p}$

$A=\frac{s+p}{s}\times P$. What is the multiplying power of a shunt? Since the fraction $\frac{s+p}{s}$ is frequently called "the multiplying power of a shunt," that is, the quantity that the current flowing through the patient must be multiplied by to obtain the total current.

As an example of the last equation, let us suppose that we desire that P shall be $\frac{1}{10}$ of A, then $\frac{s}{s+p}$ equals $\frac{1}{10}$ or s equals $\frac{1}{9}$ of p.

It would, of course, be possible to substitute for the two resistances, the patient and the shunt, (s and p) which are in parallel, a single wire of x resistance, such that *for the same potential difference*, V, *at its terminals*, the current flowing through it should be equal to the sum of the currents flowing through the two parallel circuits. To find x we have the current that would flow through it equal to $\frac{V}{X}$, then the current flowing through s equals $\frac{V}{S}$, the current flowing through p equals $\frac{V}{p}$. Therefore, since $\frac{v}{x} = \frac{v}{s} + \frac{v}{p}$, x equals $\frac{s\,p}{s+p}$, or, if two wires be parallel, then the product of their resistances, divided by their sum, represents the resistance of a single wire through which a current will pass, equal to the sum of the currents passing through the two wires, for the same potential difference. Such a single resistance is called "*the combined resistance*," or "*the parallel resistance*," of the two.

From what has preceded we see that when P is $\frac{1}{10}$ of A, $\frac{s\,p}{s+p}$ equals $\frac{1}{10}$ P, or the combined resistance of the shunt and patient is $\frac{1}{10}$ of the resistance of the patient.

In the same way, if there be any number of shunts, a, b, c, d, etc., in parallel, and x be a single resistance, that, *with the same potential difference* at its terminals, the current that will flow through x is equal to the sum of the currents that flow through all the resistances, a, b, c, d, etc, the *combined resistance* x equals

$$\frac{1}{1\text{–}a, + 1\text{–}b, + 1\text{–}c, + 1\text{–}d, \text{ etc.}}$$

The insertion of a shunt diminishes the resistance of a circuit from p to $\frac{s\,p}{s+p}$. In some cases this produces, practically, no effect on the current, so that the current flowing through the patient will be $\frac{s}{s+p}$ of the current that *was* flowing through it

*before the insertion of the shunt.* But in other cases this variation of the resistance in the circuit materially affects the total current, so that, although P is always $\frac{s}{s+p}$ of the total current, this total current may be so increased by the diminution of the total resistance that the fraction $\frac{s}{s+p}$ of the now total current is practically as large as the previous total current, or in other words, shunting the patient may produce, practically no diminution in the current passing through the patient. I mention this because the conditions we are associated with are such that this item is of great importance.

It is easily demonstrated (see Ayerton) in this way. B is a battery of 6 cells in series arranged with a cell selector. M is a galvanomotor of very low resistance. $R_1$, $R_2$, $R_3$, $R_4$, resistance coils in the main circuit. P is a galvanomotor of, say, 500 ohms resistance, also in the main circuit, bnt fitted with a shunt s. Any one of the coils, or all, can be cut out with a switch.

*Figure* 9.

The resistance in the shunt S can be varied by moving its lever. Then it is found that if the resistance in the main circuit is fairly large, say 1,000 ohms, alternating the resistance of s alters the deflection of P, but does not sensibly alter that of M; while on the other hand, if the resistance in the main circuit is small, that is, if the four resistances, R, are cut out then the value of s may be altered within wide limits without altering the value of the deflection of P. But the deflection of P will be large when the resistance in s is small, and small when the resistance in s is large.

The mathematical working out of this condition is quite simple, but I will not take the time to give the equation here. If any of you contemplate experimenting with shunts for use in cataphoresis, you will find this the foundation principle on which to build your apparatus. For in the patient, which would be represented by P in the last diagram, we have a very high resistance, and a widely-varying one. Consequently, we have the condition of S for the shunt in the exaggerated condition of all of R cut out. This has brought out the ingenious devices for varying the quantity of R and transferring part of it into the circuits of s or p in various relations. In this last illustration think of P as the patient.

Now, this would not raise a new problem in ordinary electric controllers, but in the very peculiar and complicated conditions with which we contend in the patient, it does, since we are increasing, to a certain extent, the total resistance of the circuit in which the patient is, thereby causing increased pain by any variation of potential, since the potential is higher, which was clearly demonstrated with the frog's leg. I believe that no question of electro-therapeutics has ever arisen involving so many complications of electro-physics and physiology, and it will be equally impossible for the electrician or the therapeutist to work it out alone.

## Figures and Tables

**Figure 9. f1:**